# Volumetric parameters of the primary tumor and whole-body tumor burden derived from baseline ^18^F-FDG PET/CT can predict overall survival in non-small cell lung cancer patients: initial results from a single institution

**DOI:** 10.1186/s41824-022-00158-x

**Published:** 2022-12-28

**Authors:** Hemat A. Mahmoud, Walaa Oteify, Hussein Elkhayat, Ahmed M. Zaher, Taha Zaki Mohran, Nesreen Mekkawy

**Affiliations:** 1 Department of Clinical Oncology and Nuclear Medicine, Faculty of Medicine, Assiut University, Asyût, Egypt; 2 Nuclear Medicine Department, National Cancer Institute, Cairo University, Cairo, Egypt; 3 Cardiothoracic Surgery Department, Faculty of Medicine, Assiut University, Asyût, Egypt

**Keywords:** Overall survival, Whole-body tumor burden, 18F-FDG PET/CT, Non-small cell lung cancer

## Abstract

**Background:**

Metabolic tumor volume (MTV) and total lesion glycolysis (TLG) are volumetric parameters derived from ^18^F-FDG PET/CT, suggested to have a prognostic value in cancer patients. Our study aimed to test whether these volumetric parameters of the primary tumor and whole-body tumor burden (WBTB) can predict overall survival (OS) in non-small cell lung cancer (NSCLC) patients.

**Materials and methods:**

Thirty biopsy-proven NSCLC patients who had not begun anti-tumor therapy were included in this prospective study. A baseline ^18^F-FDG PET/CT study was acquired. Scans were interpreted visually and semi-quantitatively by drawing a 3D volume of interest (VOI) over the primary tumor and all positive lesions to calculate metabolic, volumetric parameters, and WBTB. The PET parameters were used to stratify patients into high- and low-risk categories. The overall survival was estimated from the date of scanning until the date of death or last follow-up.

**Results:**

At a median follow-up of 22.73 months, the mean OS was shorter among patients with higher tu MTV and tu TLG and high WBTB. High WB TLG was independently associated with the risk of death (*p* < 0.025). Other parameters, e.g., SUV_max_, SUV_peak_, and SUV_mean_, were not predictive of outcomes in these patients. Conclusion: In patients with NSCLC, tu MTV, tu TLG, and WBTB determined on initial staging ^18^F-FDG PET/CT seems to be a strong, independent imaging biomarker to predict OS, superior to the clinical assessment of the primary tumor. The WB TLG was found to be the best predictor of OS.

## Background

Lung cancer has the highest mortality rate globally (Sung et al. 2021). More than 85% of lung cancer cases are non-small cell lung cancer (NSCLC), and adenocarcinoma is the most prevalent histological subtype (Bousquet Mur et al. 2020). The primary curative treatment for NSCLC is surgical excision, which is generally regarded as the best treatment option for patients in the early stages. Unfortunately, many NSCLC patients have missed the chance for surgery or are not candidates for radiation, targeted therapy, or immunotherapy (Shea et al. 2016). Therefore, conventional cytotoxic chemotherapy continues to be the mainstay of care for people with advanced NSCLC (Shea et al. 2016).

Currently, tumor staging is the primary factor used to determine NSCLC patients’ prognosis (Detterbeck 2018; Zappa and Mousa 2016). However, patients with the same TNM stage and identical treatment regimens have quite different tumor biology and survival rates, indicating that staging alone cannot provide sufficient clinical information (Woodard et al. 2016; Xie et al. 2018). Thus, it is necessary to identify additional discriminative prognostic markers that will improve stratification, guide tailored suitable therapy, and provide more precise forecasts of treatment outcomes and survival (Chang et al. 2019).

The guidelines include the use of ^18^F-FDG PET/CT for the diagnosis, staging, restaging, and evaluation of treatment response in lung cancer (Liu et al. 2016). FDG PET/CT functional imaging is preferred to morphological imaging, such as CT because tumor load is easier and quicker to measure (Eude et al. 2022). While maximum standardized uptake value (SUV_max_) reflects the metabolic status of the tumor, metabolic tumor volume (MTV) and total lesion glycolysis (TLG) are parameters that reflect both metabolic and volumetric status (Ventura et al. 2020). High MTV and TLG levels in lung cancer have been suggested to be prognostic factors for disease-free survival (DFS) and overall survival (OS) (Im et al. 2015). PET/CT whole-body tumor burden (WBTB), as a measure for overall burden of cancer, has been shown to bear a strong correlation with prognosis. Software developments and the growing accessibility of positron-emitting radiopharmaceuticals have allowed for considerable advancements in WBTB determination over the past ten years. However, it is still difficult to accurately estimate tumor burden, particularly when the metastatic disease is extensive (Santos et al. 2022).

### Patient selection

Between May 2019 and June 2022, 63 patients with pathologically or cytologically confirmed diagnoses of primary NSCLC were referred for whole-body 18F-FDG PET/CT scans at the nuclear medicine unit of Assiut University in Egypt. Thirty individuals were eligible to participate in our study after the exclusion of 12 patients who had begun anti-tumor therapy and 21 patients who had finished their therapy regimens and were referred for the detection of residual/ recurrent disease. All included patients were PET/CT staged according to the National Comprehensive Cancer Network guidelines. Patients with (squamous cell carcinoma, adenocarcinoma, large cell carcinoma, and poorly differentiated) NSCLC were eligible for inclusion, while, SCLC, sarcoma, neuroendocrine carcinoma, and those patients who had undergone neoadjuvant therapy before PET/CT examination, died from other diseases, or lost follow-up were excluded.

Clinical patient characteristics including gender, age, smoking status, histological type and pathological tumor grade, surgery status, and chemotherapy regimen were included. The median duration of follow-up was 17.23 months (ranging from 4 to 36 months).

### Acquisition of PET/CT

FDG data were acquired using a whole-body PET/CT scanner (Biograph, Horizon 16; Siemens, Germany). For each patient, plasma glucose levels were measured (mean Blood glucose was 6.2 ± 1.5 mmol/l) and all patients fasted for at least 6 h before scanning. PET data were acquired 60–90 min after intravenous administration of ^18^F-FDG (mean Dose was 259 ± 59.94 MBq). The scan range started at the mid-thighs and proceeded to the head. A whole-body unenhanced CT scan was performed using the following parameters: 140 kV, 150 mA, 0.8 s/rotation, 22.5 mm/s table speed, and slice thickness of 3.75 mm. Data from the CT scans were reconstructed from a 512 × 512 matrix to a 128 × 128 matrix to satisfactorily match the PET data and allow image fusion. The PET image data sets were reconstructed using an iterative algorithm (the ordered subsets expectation maximization).

### Image analysis

Two nuclear medicine physicians (with four and fourteen years of post-residency experience) examined each patient's tumor foci. The junior nuclear medicine specialist first evaluated the images, which were then corrected by the senior consultant, who then verified the final report. The primary lesion was identified by visual observation in Syngo_._via Workstation and the region of interest (ROI) was outlined around the lesion by surrounding the entire lesion with a threshold of 40% SUV_max_. The relevant semi-quantitative metabolic parameters for quantifying ^18^F-FDG uptake values were subsequently derived from the software by automatically segmenting the lesion volumetrically in the cross-sectional, coronal, and sagittal planes. The maximum standardized uptake value normalized to body mass (SUV_max_), using a SUV_max_ of 5 g/ml threshold level to view the PET images. SUV_max_, SUV_mean_, and MTV were obtained from 3D isocontour at 40% of the maximal pixel value. TLG was calculated according to the following formula: TLG = SUV_mean_ × MTV.

**Fig. 1:**
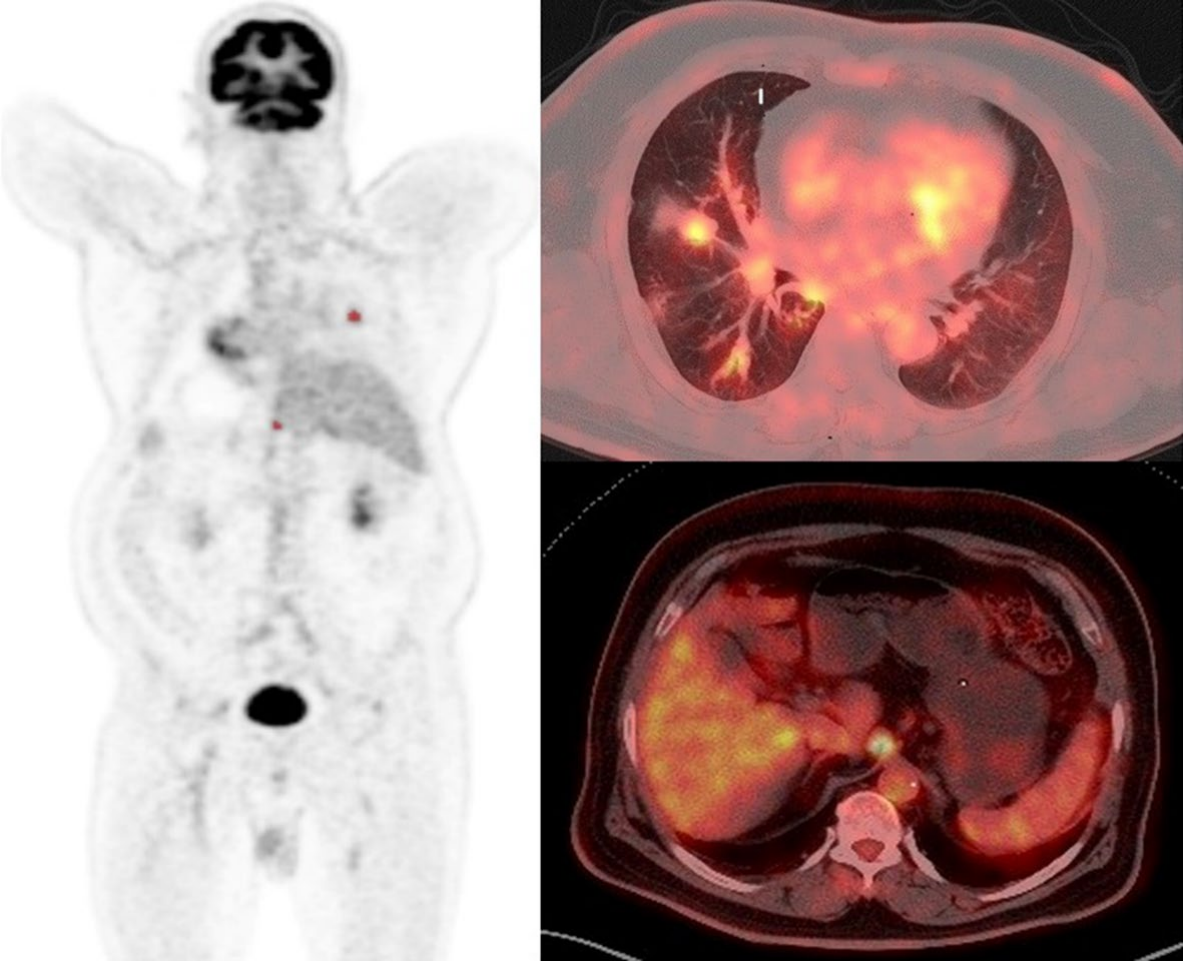
Representative ^18^F-FDG PET-CT MIP (maximum intensity projection) and axial images of a patient with low whole-body tumor burden (WBTB). A 62-year-old male patient with pathologically proven right middle lobe NSCLC, adenocarcinoma. FDG uptake was increased abnormally corresponding to the right lung primary lesion, another focal FDG uptake is noted at abdominal LN. Corresponding SUVmax, SUVpeak, SUVmean, tu MTV, tu TLG, WB MTV, and WB TLG were 3.48, 2.71, 2.04, 5.23, 10.56, 30.8, 56

**Fig. 2:**
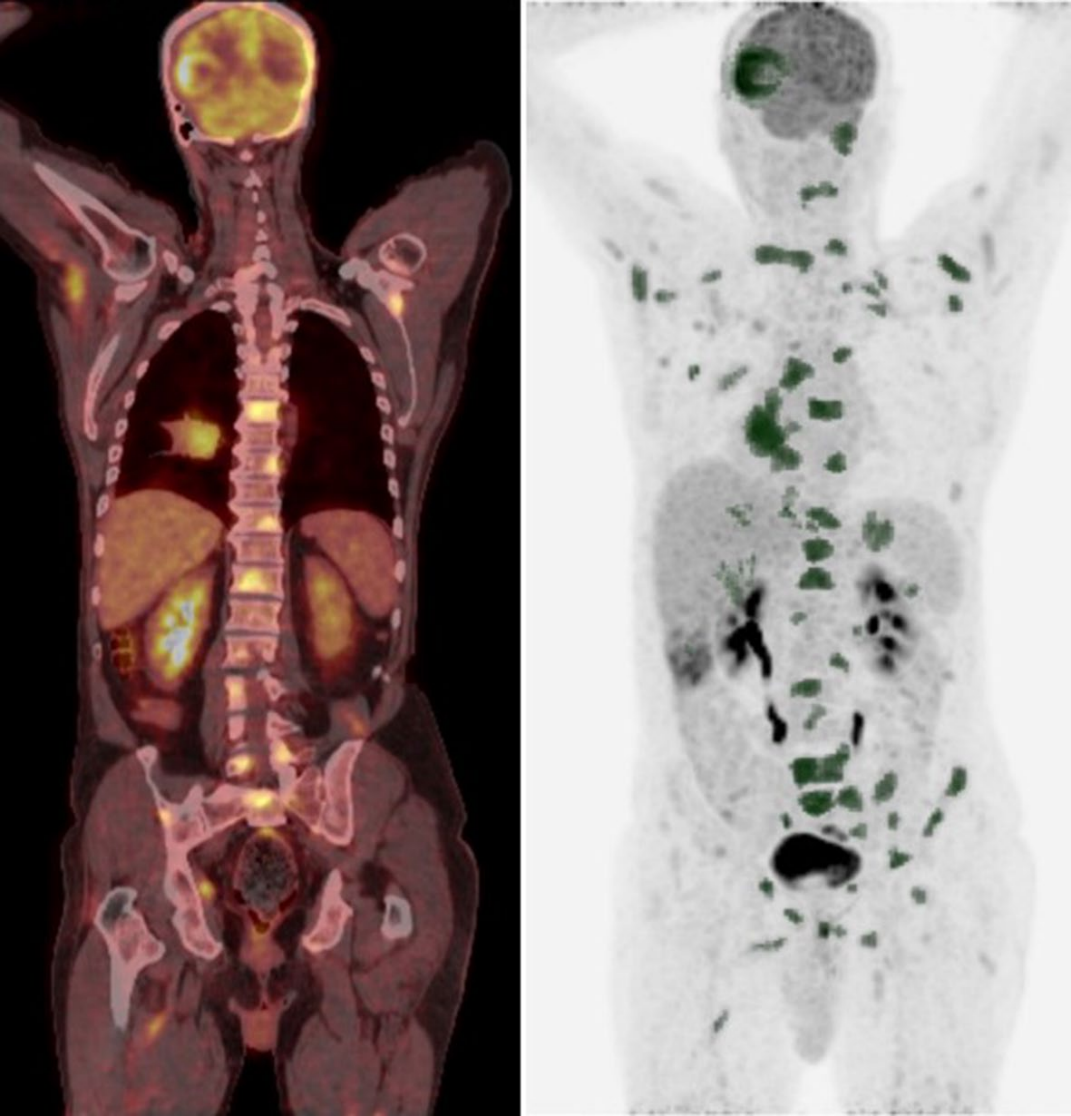
Representative ^18^F-FDG PET/CT MIP and coronal images for a patient with a high whole-body tumor burden (WBTB). A 61-year- old male patient with pathologically proven right lower lobe NSCLC, adenocarcinoma. FDG uptake was abnormally increased at the primary tumor mass as well as multiple metastatic nodal, osseous brain, and muscular deposits, corresponding SUVmax, SUVpeak, SUVmean, tu MTV, tu TLG, WB MTV, and WB TLG 4.9, 4.2, 2.9, 33, 96.03, 317.53, 931.88, respectively

The whole-body metabolic tumor volume (WB MTV) and whole-body total lesion glycolysis (WB TLG) estimated from ^18^F-FDG PET images, including primary tumors, nodes, and metastases using free LIFEx software version 7.1 (www.lifexsoft.org). Tumor volume delineation is refined using two criteria in combination, including: absolute SUV threshold (SUV > 2.5) and percent SUV_max_ threshold (41%). Then, manually removing the brain, heart, and other areas with physiological uptake. Manual adjustments were made to regions that are close to or overlap high physiological uptake regions, such as lesions in the brain or near the urinary bladder. The WB MTV and WB TLG values were automatically updated during these operations. All regions were displayed using axial, sagittal, and coronal slices and using a maximum-intensity-projection (MIP) representation. The results are exported in an Excel file and in the form of an image booklet displaying the regions. Figures 1 and 2 are representative of two patients being treated for small and large tumor volumes. Whole-body MTV was obtained by the sum of all lesions considered related to cancer and the WB TLG was obtained as the sum of the TLG values of all lesions.

Overall survival (OS) was defined as the time from the date of initial PET/CT to the date of death. Several recent studies defined overall survival as the time from enrollment in the study (initial PET/CT imaging) (Dolan et al. 2020; Peng et al. 2022; Rocha et al. 2021). The patients last known to be alive, were censored at the date of the end of the study (August 2022).

### Statistical analysis

Data were analyzed using the IBM SPSS Statistics (Version 26.0. Armonk, NY: IBM Corp). Descriptive data were shown as number and percentage values in categorical data, and mean values in continuous data. Survival receiver–operating characteristic curve analysis (ROC curve) was used to achieve the maximal area under the curve (AUC) and the optimal cutoff value for each of PET/CT-derived metabolic parameters. The PET/CT metabolic parameters were divided into high-risk and low-risk groups according to the cutoff value derived from the survival ROC. Univariate analysis of prognostic factors for OS was achieved using the Kaplan–Meier method, and the log-rank test was used to evaluate the significance of the differences between the survival curves, the Cox proportional hazards model that included significant univariate variables was used to determine independent prognostic factors for OS in multivariate survival analyses. Risk of death was estimated based on hazard ratios and the 95% confidence interval and was recorded. *P* < 0.05 was accepted for statistical significance in all analyses.

## Results

### Clinical and pathological characteristics

During the study time frame, Thirty NSCLC patients who had no prior anti-tumor therapy and planned to start first-line chemotherapy (CTH) were included. Table 1 lists the clinical features of the patients. The mean age was 63.6 ± 10.82 years, with a majority of men (73.3%). Fifteen patients (50%) had adenocarcinoma, 5 patients (16.7%) had squamous cell carcinoma, 2 patients (6.7%) had large cell carcinoma, and 8 patients (26.6%) were NSCLC of a type that was not further specified. All patients were treated with CTH alone (83.3%) while (16.7%) received CTH after surgical resection.

**Table 1:** Clinicopathological Characteristics of the Studied Patients

Variables		*N* (%)
Age	≤ 60	9 (30.0%)
	> 60	21 (70.0%)
Gender	Female	8 (26.7%)
	Male	22 (73.3%)
Smoking history	Former smoker current	20 (66.7%)
	Smoker	4 (13.3%)
	Non-smoker	6(20%)
Specific type of the NSCLC	Adenocarcinoma	15 (50%)
	Squamous	5 (16.7%)
	Large cell	2 (6.7%)
	NOS	8 (26.6%)
Tumor grade	Moderate	12 (40%)
	Undifferentiated	8 (26.7%)
	Undetermined tumor grade	10 (33.3%)
*T stage*		
T1	2 (6.7%)	
T2	9 (30%)	
T3	4 (13%)	
T4	15 (50%)	
*N stage*		
N0	4 (13.3%)	
N1	5 (16.7%)	
N2	12 (40%)	
N3	9 (30%)	
*M stage*		
M0	14 (46.7%)	
M1a	7 (23.3%)	
M1b	2 (6.7%)	
M1c	7 (23.3%)	
Clinical Stage	Stage I/II (32.5%) Stage III (26.7%) Stage IV (40.8%)	
Chemotherapy alone	26 (83.3%)	
Surgical resection with chemotherapy	4 (16.7%)	

	Mean ± SD	M (min–max)
Overall survival (months)	17.32 ± 10.73	14.5(4–36)
SUV_max_	11.19 ± 6.60	11.30 (1.20–31.80)
SUV_mean_	6.8 ± 3.89	6.6 (0.7–18.5)
Primary MTV	41.32 ± 39.90	23.65(3.7–152.7)
Primary TLG	306.07 ± 316.35	190 (1.2–1011)
WB MTV	185.45 ± 289.28	141.99 (3.09–1513.18)
WB TLG	1184.47 ± 205.01	800.97 (5.75–9671.85)

**Fig. 3:**
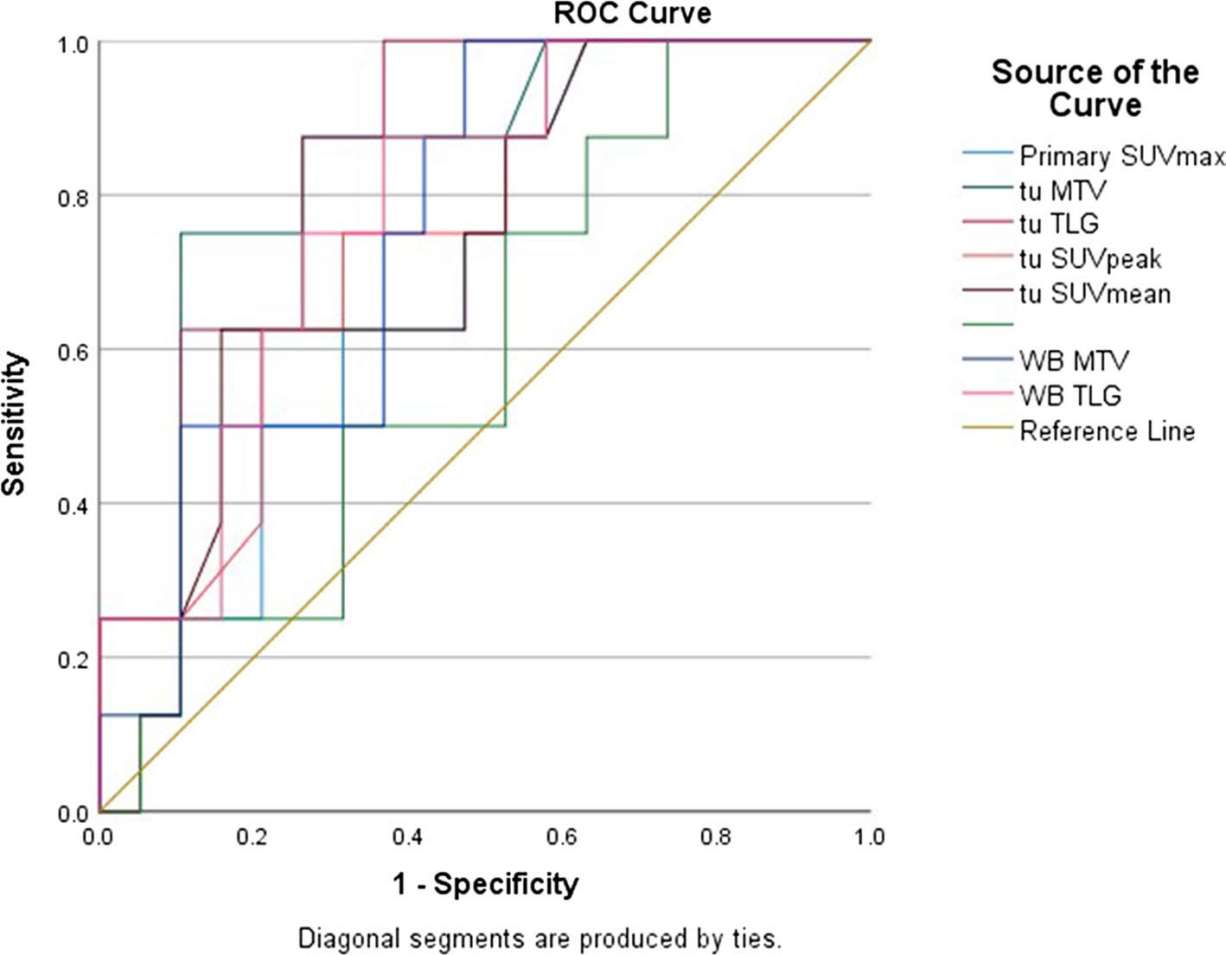
ROC curve of the predictors of overall survival

To employ metabolic and volumetric parameters of FDG PET, we dichotomized the variable with a calculated cutoff point. The cutoff point chosen based on ROC curve analysis with area under curve were: 11.3 (0.688), 9.55 (0.730),8.05 (0.724), 30.9 (0.845), 190 (0.849), 141.98 (0.757), and 832.4 (0.783) SUV_max_, SUV_peak_, SUV_mean_, tu MTV, tu TLG, WB MTV, and WB TLG, respectively. The results of ROC analysis of PET prognostic markers are presented on Table 2 and Fig. 3

**Table 2:** ROC analysis, AUC, and Optimal Cutoff Value of Each Metabolic Parameter

	AUC	*P* value	95% CI		Cut off point	Sensitivity (%)	Specificity (%)
			Lower	Upper			
Primary SUV_max_	.688	0.130	.485	.890	> 11.30	75.0	52.6
Primary SUV_peak_	.845	0.063	.538	.923	> 9.55	74.0	68.4
Primary SUV_mean_	.849	0.071	.523	.924	> 8.05	62.5	84.2
Tu MTV	.730	0.005*	.687	1.000	> 30.9	87.5	73.7
Tu TLG	.724	0.005*	.704	.993	> 190.0	87.5	61.2
WB MTV	.757	0.038*	.572	.941	> 141.98	87.5	57.9
WB TLG	.783	0.022*	.605	.961	> 832.4	87.5	63.2

*ROC* Receiver operating characteristic curve, *AUC* Area under the curve, *CI* Confidence interval, *MTV* Metabolic tumor volume, *TLG* Total lesion glycolysis, *WB MTV* Whole-body metabolic tumor volume, *WB TLG* Whole-body total lesion glycolysis,

Bold are highlighted the p values that reached statistical significance is P value < 0.05

### Kaplan–Meier survival analysis

**Fig. 4:**
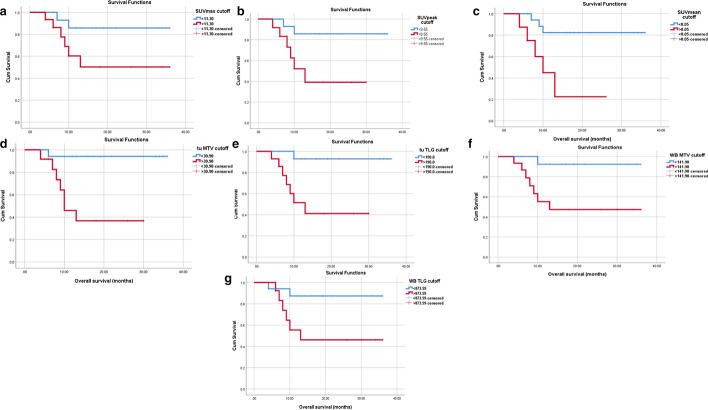
Kaplan–Meier curves of overall survival (OS) stratified according to dichotomized PET/CT parameters. **a** SUVmax, **b** SUVpeak, **c** SUVmean, **d** tu MTV and **e** tu TLG **f** WB MTV **g** WB TLG in all patients (n = 30). The blue lines indicate the group with values less than the cutoff values and the red lines are the group with values equal to or greater than the cutoff values

The probability of survival above or below the calculated cutoff points of different variables was calculated. The results of univariate survival analysis are presented in Table 3. High tu MTV and tu TLG as well as WB MTV and WB TLG were associated with significantly shorter OS. Mean OS for MTV ≥ 30.9 mL versus MTV < 30.9 mL was 16.56 months (95% CI: 14.532–30.31) versus 34 months (95% CI: 27.022–37.121) *(P* = *0.006).* Mean OS for TLG ≥ 190 was 17.31 months (95% CI: 11.088–23.5) versus 33.83 months for TLG < 190 g (95% CI: 29.77–37.89) *(P* = *0.008).* For WB MTV the mean OS was 21.38 months for values ≥ 141.98 (95% CI 13.82–28.94) versus 34 months for values < 141.98 (95% CI: 30.234–37.76) *(P* = *0.011).* Mean OS for WB TLG ≥ 832.4 g was 20.17 months (95% CI: 12.35–27.98) vs 34 months for WB TLG < 832.40 g (95% CI 30.23–37.76) (*P* = *0.007).* See Fig. 4 for Kaplan–Meier curves based on SUV_max_, SUV_peak_, SUV_mean_, tu MTV, tu TLG, WB MTV and WB TLG.

**Table 3:** Mean overall survival values of high and low-risk groups categorized according to different PET parameters

Variable	Category	Event/Total	Percentage of censored (%)	Median survival (months)
SUV_max_	< 11.30	2/15	86.7	32.07
	> 11.30	6/15	60.0	22.42
SUV_peak_	< 9.55	2/16	87.5	32.07
	> 9.55	6/12	50	17.06
SUV_mean_	< 8.05	3/20	85	31.18
	> 8.05	5/8	37.5	12.72
tu MTV	< 30.9	1/18	94.5	34
	> 30.9	7/12	41.7	16.55
tu TLG	< 190	1/16	93.75	33.833
	> 190	7/14	50	17.30
WB MTV	< 1990	1/14	92.86	34.00
	> 1990	7/16	56.25	21.39
WB TLG	< 832.40	2/17	88.23	34.00
	> 832.40	6/13	53.84	20.17

Mean OS for high SUV_max_ was shorter than for low SUV_max_, but the difference was not significant. Mean OS for SUV_max_ < 11.3 versus SUV_max_ ≥11.3 was 32.07 months (95% CI: 27.02-37.12) versus 22.42 months (95% CI: 14.53- 30.31) (*P* = 0.054). Mean OS for SUV_peak_ < 9.55 versus SUV_peak_ ≥ 9.55 was 32.07 months (95% CI: 27.022-37.12) versus 17.05 months (95% CI 10.31–23.8) (*P*= 0.018). Mean OS for SUV_mean_ < 8.05 versus SUV_mean_ ≥ 8.05 was 31.18 months (95% CI 26.21-36.13) versus 12.73 months (95% CI: 6.58-18.) *(P*= *0.005).* (Table 3).

Regarding clinical and pathological factors, patients with squamous cell pathological subtype had lower mean OS *(P* = *0.002).* Otherwise, no other clinical factors of age, sex, smoking history were predictors of OS (P value = 0.4, 0.3, and 0.2, respectively).

### Cox univariate and multivariate analyses

The previous cutoffs were used to divide the study population into 2 distinct prognostic groups for Kaplan–Meier and Cox survival analysis. Multivariate Cox regression analysis showed that tu MTV (hazard ratio of 1.017, 95% CI:1.003–1.031, *p* = *0.019*), tu TLG (hazard ratio of 1.002, 95% CI:1.000–1.004, *P* = *0.023*) and WB TLG (hazard ratio of 1.001, 95% CI:1.000–1.002, *p* = *0.005*) were significant in multivariate analysis for OS.

WB TLG was confirmed as an independent predictor. High WB TLG patients had higher risk of death than low WB TLG patients, with an adjusted hazard ratio of 1.001 (95% CI:1.000–1.002, *P* = *0.025*). Parameters that independently affect OS in the COX regression analysis are shown in Table 4

**Table 4:** Multivariate Cox regression on the association of metabolic and volumetric parameters with OS

	Univariable Cox analysis	95% C.I		*P* value	Multivariable Cox analysis	95% C.I		*P* value
	HR	Lower	Upper		HR	Lower	Upper	
Age	1.039	0.961	1.123	0.334				
Gender (male)	2.707	0.332	22.065	0.352				
SUVmax	1.069	0.984	1.162	0.115				
SUV peak	1.088	.985	1.203	0.097				
SUV mean	1.121	.971	1.293	0.118				
Tu MTV	1.017	1.003	1.031	**0.019**	0.960	0.896	1.030	0.254
Tu TLG	1.002	1.000	1.004	**0.023**	1.006	0.998	1.015	0.158
WB MTV	1.001	1.000	1.003	0.060				
WB TLG	1.001	1.000	1.002	**0.005**	1.001	1.000	1.002	**0.025**

In bold are highlighted the *p* values that reached statistical significance

## Discussion

NSCLC management is a primary concern, therefore prognostic assessment is critical to realizing the potential of personalized treatment. A wide range of information sources are available of which the noninvasive type plays a fundamental role in reducing the patients' burden (Lambin et al. 2015). As a noninvasive imaging modality, the ^18^F-FDG PET/CT, which reflects tumor metabolic activity, such as cell viability and proliferative activity, is becoming more and more significant in studies of initial diagnosis, accurate staging, evaluation of treatment response, and prognosis of lung cancer (Li et al. 2019).

In this prospective study, we hypothesized that the metabolic parameters derived from ^18^F-FDG PET/CT may provide more accurate quantitative imaging features and thus might be of value in predicting OS in patients with NSCLC.

In PET/CT imaging, the SUV is the most widely used semi-quantitative measure. The individuals' physical characteristics, the blood glucose level, the uptake period, the image acquisition and reconstruction, the lesion size, and other variables all have an impact on SUV (Lee et al. 2012).

SUV_max_, SUV_mean_, and SUV_peak_, represent the maximum, average, and peak SUV of the region of interest, respectively. All three of these parameters were measured and examined in our study in order to determine which was the most valuable. We found that higher levels of SUVpeak and SUVmean were associated with shorter OS which was not true for SUVmax. Our findings further support the hypothesis that SUVmax could not be a reliable predictor of OS, emphasizing the significance of volumetric parameters. Given that SUV_max_ simply represents a single-pixel value of peak metabolic activity, hence, has less impact on patient’s outcome, Huang and colleagues suggested that SUV_max_ may not be the most reliable predictor (Huang et al. 2014).

Increasing evidence from studies on different tumor types shows that MTV and TLG are more accurate prognostic indicators than do SUV metrics (Hyun et al. 2014; Liao et al. 2012).

Several automated techniques are used to segment regions of interest in PET/CT scans, including gradient-based threshold (adaptive iterative algorithm, AIA), fixed SUV threshold (e.g., SUV > 2.5), percentage threshold of SUV_max_ (e.g., > 42%), and background-related threshold (AT 40%) approaches. The fixed threshold method was not used in our study since it ignores the background activity as reported in earlier studies (Soret et al. 2007). Percentage threshold can be performed rapidly and consistently, with less inter-observer variability (Wang et al. 2017). In our study we used percentage threshold method for delineation of MTV. Our results reported a significantly worse OS and higher risk of death in patients with higher tu MTV and tu TLG. These results are in line with MTV's established predictive value in previous studies (Popinat et al. 2019; Seban et al. 2020; Jreige et al. 2019).

The definition of "high tumor burden" is still up for debate. Its definition and use as a prognostic factor in the context of lung cancer could assist in the development of specialized treatment regimens that would not only enhance patient quality of life and treatment outcomes but also lower the occurrence of negative adverse effects (Reck and Rabe 2017).

Although earlier studies have employed the MTV methodology (with a threshold value of 41% of SUVmax in each lesion), more advanced methods might yield more precise measurement of WB MTV values. Chardin et al. reported that WB MTV, as calculated by threshold value method, offers a reliable and reproducible estimate of whole-body tumor burden (Chardin et al. 2020).

The optimal cutoff value of WB MTV in our study was 141.98 ml. Due to the lack of current standard ^18^F-FDG PET/CT parameters cutoff definitions, external validation of the results may be challenging. The WB MTV cutoff values published in research to date have varied, while utilizing comparable methods to compute MTV. These values range from 17.8 to 143.2 ml (Hashimoto et al. 2020; Dall'Olio et al. 2021; Monaco et al. 2021; Yamaguchi et al. 2020). Pu et al. validated WB MTV through analysis of a quite large sample of NSCLC patients of all clinical stages reporting outcomes comparable to ours (Pu et al. 2018). Consequently, Pellegrino and colleagues additionally found that WB MTV was a reliable predictor of OS at all TNM stages (Pellegrino et al. 2019).

In addition to WB MTV, we also assessed the other volumetric value WB TLG. Supposedly, TLG could be more promising since it combines volumetric and metabolic information. Our results found that WB TLG is an independent predictor of OS. Similar results were reported by a previous study by Vanhove whose results showed that only WB TLG is of prognostic value in NSCLC for both OS and progression free survival (PFS) (Vanhove et al. 2018).

In a recent study by Oliveira et al. 52 NSCLC patients who performed ^18^F-FDG PET/CT for staging in who were followed for a median of 11.0 months, they found that The WB TLG/tu TLG ratio is an independent prognostic indicator of OS in advanced-stage NSCLC (Oliveira et al. 2022).

A multi-institutional investigation confirms that SCC histology is independently correlated with lower overall survival and local, regional, and distant recurrence (Baine et al. 2018). This comes in agreement with our results that showed a significantly shorter OS survival of SCC. Future research is required to evaluate whether early-stage NSCLC therapy paradigms should vary based on histology.

The Cox multivariate analysis revealed that the degree of histological differentiation had no statistically significant effect on OS. Possible explanation of this result is that OS is frequently impacted by numerous other factors, such as type of therapy, tumor recurrence or metastasis. Additionally, the histological differentiation of the tumor was not determined in this study for one-third of the patients. Therefore, we were only working with 20 cases whose degree of difference was known. The statistical analysis result may be biased by a quite small sample size.

In conclusion, we have demonstrated that, in patients with NSCLC, the whole-body metabolic tumor burden determined on a baseline 18F-FDG PET/CT performed for staging seems to be a strong, independent imaging biomarker to predict OS, superior to the clinical assessment of the primary tumor itself. The most accurate predictor of OS in our patients was the WB TLG. Efforts should be made to unify its definition and to further explore its potential as a prognostic factor for patients with metastatic NSCLC.

Our study was limited by the small number of study group and short follow-up period. This may be attributed to COVID-19 pandemic. According to a survey conducted online by the International Atomic Energy Agency on a variety of nuclear medicine services and completed by 434 doctors from 72 different countries, nuclear medicine services have dramatically declined, by over 50% for diagnostic tests and as much as 45% for radionuclide therapy (Freudenberg et al. 2020).

Other limitations include the heterogeneity of the study population considering the various NSCLC subtypes, various metastatic statuses, and different previous therapies.

Our benefits were a single-center research design, patient preparation, waiting period, and FDG doses at the stage preceding FDG PET/CT were uniform, the same FDG PET /CT scanner for all patients.

## Data Availability

All data used in this study can be available on request.

## Abbreviations

OS: Overall survival
NSCLC: Non-small cell lung cancer
tu MTV: Metabolic tumor volume of the primary tumor
tu TLG: Total lesion glycolysis of the primary tumor
WBTB: Whole-body tumor burden
WB MTV: Whole-body metabolic tumor volume
WB TLG: Whole-body total lesion glycolysis
